# Predicting ACL Injury Using Machine Learning on Data From an Extensive Screening Test Battery of 880 Female Elite Athletes

**DOI:** 10.1177/03635465221112095

**Published:** 2022-08-19

**Authors:** Susanne Jauhiainen, Jukka-Pekka Kauppi, Tron Krosshaug, Roald Bahr, Julia Bartsch, Sami Äyrämö

**Affiliations:** † Faculty of Information Technology, University of Jyväskylä, Jyväskylä, Finland; ‡ Oslo Sports Trauma Research Center, Department of Sports Medicine, Norwegian School of Sport Sciences, Oslo, Norway; Investigation performed at University of Jyva«Â€skyla«Â€, Jyva«Â€skyla«Â€, Finland

**Keywords:** predictive methods, machine learning, prediction significance, cross-validation, motion analysis, ACL injury, team sports

## Abstract

**Background:**

Injury risk prediction is an emerging field in which more research is needed to recognize the best practices for accurate injury risk assessment. Important issues related to predictive machine learning need to be considered, for example, to avoid overinterpreting the observed prediction performance.

**Purpose:**

To carefully investigate the predictive potential of multiple predictive machine learning methods on a large set of risk factor data for anterior cruciate ligament (ACL) injury; the proposed approach takes into account the effect of chance and random variations in prediction performance.

**Study Design:**

Case-control study; Level of evidence, 3.

**Methods:**

The authors used 3-dimensional motion analysis and physical data collected from 791 female elite handball and soccer players. Four common classifiers were used to predict ACL injuries (n = 60). Area under the receiver operating characteristic curve (AUC-ROC) averaged across 100 cross-validation runs (mean AUC-ROC) was used as a performance metric. Results were confirmed with repeated permutation tests (paired Wilcoxon signed-rank-test; *P* < .05). Additionally, the effect of the most common class imbalance handling techniques was evaluated.

**Results:**

For the best classifier (linear support vector machine), the mean AUC-ROC was 0.63. Regardless of the classifier, the results were significantly better than chance, confirming the predictive ability of the data and methods used. AUC-ROC values varied substantially across repetitions and methods (0.51-0.69). Class imbalance handling did not improve the results.

**Conclusion:**

The authors’ approach and data showed statistically significant predictive ability, indicating that there exists information in this prospective data set that may be valuable for understanding injury causation. However, the predictive ability remained low from the perspective of clinical assessment, suggesting that included variables cannot be used for ACL prediction in practice.

Anterior cruciate ligament (ACL) injuries are a major concern in team and cutting sports, making injury prevention essential and prediction alluring.^33,38^ However, while multiple potential risk factors have been suggested in the literature, whether a future ACL injury can be predicted is still a matter of controversy. Advances in data collection and storage, as well as computational power, have opened new possibilities, but there are several potential pitfalls and, consequently, also a number of important guidelines to consider to obtain reliable and valid results. The main pitfall is confusion around what is actually considered prediction in sports injury research and the difference between explanatory and predictive analyses.

Sports injury research has mainly been based on traditional statistical inference^43^ with a focus on explaining or understanding phenomena of interest in the data sample at hand. This approach is also referred to as explanatory analysis.^5,45^ The boundary between explanatory analysis and machine learning (ML) is not at all unambiguous, but in ML, the generalizability of a model usually takes precedence over its explainability. Generalizability means the ability to make accurate predictions on new unseen observations, and this approach is also referred to as predictive analysis.^4,45^ Predictive analysis requires testing generalizability on carefully selected independent (test) data (ie, examples not involved in model fitting or selection).

Several injury prediction studies have been conducted in the past using biomechanical data in combination with, for example, anthropometrics and strength measurements.^17,33,34^ However, these studies have several limitations, making their validity questionable. First, they predict knee abduction moments as a surrogate for injury based on the assumption that high knee abduction moments predict ACL injury risk. This assumption, however, is based on explanatory analyses of data from a pilot study with <10 injury cases,^16^ which is inadequate. Risk factors recognized in explanatory studies only demonstrate a statistical association with injuries but offer no evidence that they have predictive ability.^1,43,45^ Moreover, the biomechanical data that these models are based on originate from a vertical drop jump (VDJ) task. Other, much larger studies have shown small or no associations between biomechanics (including knee abduction moments) and injury risk in the VDJ task.^21,47^

Another important pitfall in prediction is inadequate assessment of the generalizability of the predictive models. Many ML methods have practically infinite ability to fit in complex phenomena present in the data, given sufficient computational resources. On the other hand, this high learning capacity risks overfitting, and therefore it is critical to test the generalizability of a predictive model properly before it is implemented into practice.^22^ Importantly, the role of chance results should be considered, ensuring that the predictive performance is better than chance and not just a singular random result.^18^ This is essential with small and/or high-dimensional (ie, large number of variables) data sets as well as imbalanced data, which often is the case in sports injury prediction. For example, in neuroscience the problem of chance findings has been widely recognized and permutation tests have been suggested for confirming findings.^8^ Moreover, the use of cross-validation, the most popular way to estimate model generalization ability in many fields, introduces randomness to the analysis and results can vary widely based on the fold division,^12^ as was apparent in a recent hamstring injury prediction study.^44^ An example in which these pitfalls were not considered is a recent ACL injury prediction study that did not exclude the possibility of a chance result.^52^ While their study uses predictive analysis (ie, independent test data to assess generalizability), the high test accuracy (92%) against notably lower validation accuracy (70%) strongly suggests overfitting to test data either by (unconsciously) repeatedly resampling the test data set or purely by chance.

Obviously, it is also important to consider what types of data are best for injury prediction use.^19^ No matter how appropriately the ML process is planned, no method is able to describe phenomena that are not captured in the data in the first place. Sports injury causation is multifactorial, indicating that a large number of variables, covering different properties and their interrelationships, should be considered.^25,28^ With modern computational power, ML enables efficient analysis of a large amount of data and variables, including their interactions and nonlinear relationships, and is therefore thought to have potential in most fields, including sports injury research.^40,41^ The predictive ability of previously recognized factors needs to be assessed in different settings and populations. However, periodic screening tests might not be sufficient for sports injury prediction,^1^ and thus far only a few studies exist and results are variable.^19,25,42,44^

Therefore, the purpose of this study was to investigate the predictive ability of data from a large prospective ACL injury screening study, taking into account the effect of chance results and randomness from cross-validation. We applied a recently published ML approach^19^ and extended the ML hypothesis space by applying different methods and preprocessing techniques for handling class imbalance in the data.

## Methods

### Participants

The data used in this study were originally collected for a cohort study designed to examine risk factors for noncontact ACL injuries in female elite handball and soccer players.^21,32,35,38,46,49,50^ A total of 451 soccer and 429 handball players (age, 21 ± 4 years; height, 170 ± 6 cm, weight, 66 ± 8 kg) were tested between the years 2007 and 2015. For the 2007 season, handball players with a first-team contract who were expected to play in the premier league were eligible for participation. Additionally, new players were invited for preseason testing when new teams advanced to the premier league between 2008 and 2014. From 2009, soccer players from the female premier league were also included. The study was approved by the regional committee for medical research ethics, the South-Eastern Norway Regional Health Authority, and the Norwegian Social Science Data Services, Norway. Players signed a written informed consent form before inclusion (including parental consent for players aged <18 years).

### Data Collection

**Figure 1.**
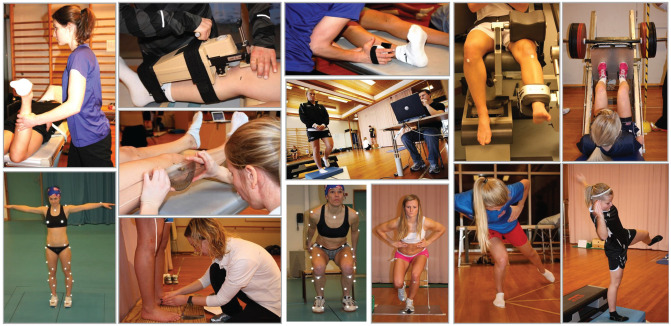
Examples of the conducted tests (hip anteversion, knee joint laxity [KT-1000], hip abductor isometric strength, quadriceps/hamstrings isokinetic strength, leg press, marker-based static anthropometric measures, knee recurvatum, single-leg balance, navicular drop/pronation, vertical drop jump, single-leg squat, star excursion test, single-leg drop stabilization).

At baseline, each player participated in a comprehensive set of screening tests designed to assess potential demographic, neuromuscular, biomechanical, anatomic, and genetic ACL injury risk factors. The screening tests were conducted at the Norwegian School of Sport Sciences in the preseason, June through August for handball and February through March for soccer. A baseline questionnaire was completed on player characteristics, elite playing experience, and history of any previous injuries to the ACL. Additionally, a variety of tests measuring anthropometrics, strength, flexibility, and balance were conducted (Figure 1). Included variables are described in Appendix Table A1 (available in the online version of this article), and for a more detailed description of the tests see Mok et al^31^ and Pasanen et al.^37^

**Figure 2.**
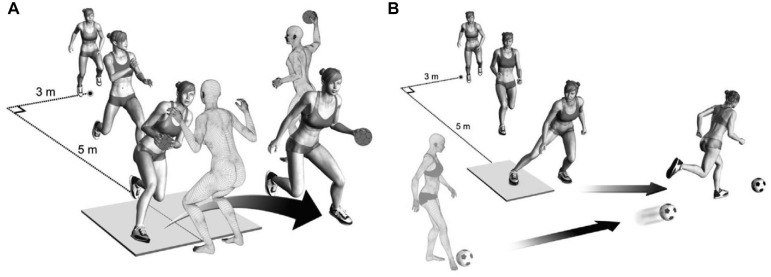
The testing situation of (A) the handball-specific sidestep cutting task and (B) the soccer-specific sidestep cutting task. Reprinted from Mok KM, Bahr R, Krosshaug T. Reliability of lower limb biomechanics in two sport-specific sidestep cutting tasks. *Sport Biomech*. 2017;17(2):157-167.^31^ Reprinted with permission of the publisher (Taylor & Francis Ltd, http://www.tandfonline.com).

Three-dimensional motion analysis was carried out on VDJ and cutting tasks. The VDJ was performed from a 30-cm box. Players were instructed to drop off the box and perform a maximal jump upon landing with their feet on 2 separate force platforms (LG6-4-1; Advanced Mechanical Technology Inc). For more details on the VDJ protocol and setup see Krosshaug et al.^21^ The sidestep cutting task was sport specific (Figure 2); the handball players performed a handball-specific faking maneuver involving a static human defender, while the soccer players performed a sidestep cutting task with a soccer through-pass. For a more detailed description of the cutting protocols see Mok et al.^31^ Full-body kinematics were captured with 35 reflective markers attached over anatomic landmarks on the legs, arms, and torso.^20^ From 2008 to 2011, 2 additional markers (left and right iliac crest) were used for those players whose markers on the left and right anterior superior iliac spine were occluded. From 2012 and onward, the crest markers were included for all players but only used in cases in which the anterior superior iliac spine markers were occluded. Between 2007 and 2012, eight 240-Hz infrared cameras (ProReflex; Qualisys) were used together with 2 force platforms collecting at 960 Hz. From 2012, an upgraded 16,480-Hz camera system (Oqus 4; Qualisys) was used. Marker trajectories were calculated and tracked with the Qualisys Track Manager. For a more detailed description of the motion data collection and variable extraction, see Krosshaug et al.

We recorded all complete ACL injuries among the tested players through May 2015, primarily through semiannual contact with the participating teams (manager, coach, medical staff). If any acute knee injuries occurring during regular team training or competition were reported, we contacted the injured player by telephone to obtain detailed medical data and a description of the injury situation. All ACL injuries were verified by magnetic resonance imaging and/or arthroscopy. The injury mechanisms were self-reported as contact (ie, direct contact to the lower extremity), indirect contact (ie, contact with other body parts), or noncontact, and these were categorized into 2 groups: noncontact/indirect contact or contact.^36^

### Data Preprocessing

All data analyses were performed with MATLAB R2018b (MathWorks Inc) and classifiers run with the *Statistics and Machine Learning Toolbox 11.0*. For the 3-dimensional motion analysis data as well as other variables with multiple trials or measurements (star excursion, hip abduction, navicular drop), a mean of trials was calculated for analyses. For generalized joint laxity, the sum of the 9 tests included was calculated. The variables that had been measured separately for the right and left legs were transformed to dominant (leg used for kicking a ball) and nondominant leg variables, and participants with missing dominance information were dropped (n = 14; 0 injured). Participants with a contact ACL injury were excluded (n = 9) to focus prediction on noncontact and indirect contact. Additionally, players with more than 50% of missing data (n = 66; 5 injured) were excluded, and finally, the data set used for analyses included 791 players with 60 ACL injuries and 283 variables.

To ensure validity of measurements, the most obvious outliers were identified with MATLAB’s *isOutlier* function, as those that were >2 scaled median absolute deviations from the variable median (see function documentation for definition). If the function indicated possible outliers, visual confirmation was done to decide whether a value was a clear mistake or measurement error in data. In this case, only that 1 value from the particular participant was discarded. Visual analysis is a common preprocessing approach, and here it ensures as little data as possible are excluded in the cleaning process. Altogether, 47 values (0.0001%) from 16 players were discarded, with only 4 values being from injured players.

After discarding outliers, 9029 missing values (4.01% of total) existed across 478 players. These were imputed with the k-nearest-neighbor (knn) imputation with a k value of 10. Knn imputation works by finding the k most similar (measured with Euclidean distance in this study) observations and imputing the missing value with a summary metric (mean used in this study) from these k similar players. For weight and height, if a measured value was missing, a linear regression approach was used to impute a value based on the self-reported values.

Continuous variables were normalized to have a mean of 0 and SD of 1 for each column, while discrete variables were centered around 0. In addition, variations in data between sport (ie, different cut test in soccer vs handball) as well as different test years, to account for potential minor differences in testing procedures, were considered in normalization by including sport and test year in addition to labels in the stratified cross-validation split and normalizing each test group separately.

### Choice of Classifiers

Four commonly used methods, random forest, L2-regularized logistic regression, and support vector machines (SVMs) with both linear and nonlinear kernel, were chosen as binary classifiers in our analyses. Random forest is a nonlinear classification and regression method that has become a standard data analysis tool in different fields such as medicine and bioinformatics^3^ and has been used in sports injury research as well.^6,19,25,44^ It is based on building an ensemble of multiple decision trees.^4^ The model (*TreeBagger* MATLAB function) was trained with a hundred trees,^4^ and Bayesian optimization^48^ with *bayesopt* function was used to select the minimum number of observations per tree leaf (from 50 to 150) and the number of predictors to sample at each split (from 1 to 100). L2-regularized logistic regression, in turn, is a linear classifier that shrinks regression coefficients by penalizing the model with the L2 norm.^13^ Regularization can discard irrelevant variables and possibly increase predictive performance and decrease overfitting of a model.^13^ It also works well with highly correlated variables.^27^ The model was trained with MATLAB’s *fitclinear* function, and the optimal amount of penalization was estimated with Bayesian optimization from the default values.

SVMs are powerful and flexible classifiers^22^ trying to find a hyperplane that best separates the classes from each other. They have previously been used to model nonlinear patterns and interactions in sports injury research.^6,44^ In this study, we trained the SVM models with the *fitcsvm* function with both linear and nonlinear (*rbf*) kernel to assess both interactions. Hyperparameters for kernel scale (as default values from 0.001 to 1000) as well as box constraint (as default values from 0.001 to 1000) were selected with Bayesian optimization.

### Data Imbalance Handling

Data imbalance means that there are clearly more observations from 1 (or more) class (majority class) than the other(s) (minority class). It is a very common and troublesome issue in the ML field,^23^ and multiple different approaches to handle data imbalance have been developed and applied, including in the sports injury prediction field recently.^10,25,43,44^

Random undersampling simply means that the majority class is limited by randomly deleting observations from it, resulting in a balanced but smaller data set. Random oversampling works similarly but instead increases the observations in the minority class by randomly duplicating them, thus making the data set larger. The Synthetic Minority Oversampling Technique (SMOTE) can be used to increase the minority class observations in a balanced way.^7^ It works by utilizing the existing minority examples as input and creates new observations by combining variables based on the knn algorithm. In cost-sensitive learning, the cost of misclassifying a minority observation is set higher than the cost of misclassifying a majority example. For example, in sports injury prediction (or medicine in general), not identifying an injury can be considered more harmful than incorrectly predicting some healthy athletes as injured. In practice, this is often achieved by providing the trained model a weight vector,^24^ in which a higher value is set for observations corresponding to the minority class.

In this study, we experimented with the effect of random undersampling, SMOTE, as well as class weight vector in the training phase on the injury prediction task. For SMOTE, a MATLAB implementation from the MATLAB Central File Exchange^26^ based on the original paper by Chawla et al^7^ was used. For training class weights, each of the used methods contains an inbuilt hyperparameter option *Weights*, and a 10 times higher cost was set for the minority class.

### Validation

In predictive analysis, a model’s generalizability to new data has to be assessed with independent test data, that is, data that have not been used in the training of the model.^22^ The most common way to do this is by splitting data into separate training and testing data or by cross-validation. K-fold cross-validation is based on randomly splitting the data into K sets and leaving each set at a time for testing while the rest of the sets are used to train a model. In general, k-fold is a common approach when data size is limited, as the complete data can be utilized for training the model.^13^ In this study, we used 5-fold cross-validation.^13^ Normalization and imputation of the training data were done separately inside each fold, and the test data were then normalized using coefficients estimated from the training data.

In addition, the model performance metric needs to be chosen carefully, especially with imbalanced data sets, which is often the case in sports injury prediction. Accuracy, for example, is not suitable with a class imbalance, as simply assigning all observations to the major class will yield high results. We assessed test performance with area under the receiver operating characteristic curve (AUC-ROC).^11^ It is based on both true-positive and false-positive rates, and it can be used with imbalanced class distributions,^11,22^ which was the case in our data. The value can be defined as excellent (0.90-1), good (0.80-0.89), fair (0.70-0.79), poor (0.60-0.69), or fail (0.50-0.59).^21,29^

### Confirmatory Data Analysis

To avoid singular chance findings and ensure that the achieved results are not just due to some noise or fluctuations in data but actually present patterns significantly above a chance level, permutation tests with multiple repetitions can be utilized.^8^ By repeating the analyses, the variation in results by cross-validation can be assessed. In practice, permutation tests are done by training a reference model, randomly shuffling the labels in the training phase, and then comparing it with the actual model trained with true labels. If the true models are consistently better than the random models across repetitions, the results are confirmed not to be observed by chance or just due to some noise in the data. In this study, the analysis was repeated a hundred times for both true and random models, and Wilcoxon signed-rank tests were used for a paired comparison to confirm the significance of achieved predictive performance.^19^ The limit of significance was set to α = .05, and in each cross-validation run, the fold divisions were kept the same for random and true models to allow fair pairwise comparison. Permutation tests were not run for the data imbalance handling analyses.

## Results

The mean AUC-ROC predictive ability was relatively consistent between the various ML methods (Table 1). Linear SVM without any imbalance handling achieved the highest mean AUC-ROC value of 0.63. For all methods, the AUC-ROC values were higher (*P* < .001) with the real responses than with the random models. With all 4 classifiers, there was a notable difference between the minimum and maximum AUC-ROC values achieved across repetitions, caused by the random cross-validation splits.

**Table 1:** AUC-ROC Values Over the 100 Repetitions^*a*^

	Logistic Regression	Random Forest	Linear SVM	Nonlinear SVM
Test	0.61 ± 0.02	0.57 ± 0.02	0.63 ± 0.02	0.61 ± 0.03
Min-max, range	0.57-0.65	0.51-0.63	0.55-0.67	0.53-0.69
Permuted	0.58 ± 0.03	0.52 ± 0.04	0.50 ± 0.04	0.49 ± 0.04
Training	0.86 ± 0.01	0.98 ± 0.01	0.96 ± 0.01	0.98 ± 0.02
SMOTE	0.60 ± 0.02	0.56 ± 0.02	0.58 ± 0.02	0.59 ± 0.02
Weighted	0.61 ± 0.02	0.58 ± 0.03	0.59 ± 0.02	0.60 ± 0.02
Undersampling	0.57 ± 0.03	0.50 ± 0.00	0.57 ± 0.03	0.58 ± 0.03

a Data are presented as mean ± SD area under the receiver operating characteristic curve (AUC-ROC), unless otherwise indicated. Permuted row correspond to the values for the random model and training row to the values for the training data. SMOTE, Synthetic Minority Oversampling Technique; SVM, support vector machine.

The training AUC-ROC values were very high with the random forest and SVMs, but with logistic regression, regularization seemed to control overfitting better. The test AUC-ROC values were, however, relatively similar despite differences in the training AUC-ROC. Additionally, preprocessing to handle class imbalances, that is, using SMOTE, class weight, and random undersampling, did not improve the prediction results, but results seemed similar or even slightly worse depending on the technique.

## Discussion

### Main Findings and Clinical Relevance

This study investigated the predictive ability of a large prospective ACL injury screening data set with 60 injury cases, using 4 common ML algorithms, repeated cross-validation runs, and permutation tests that will ensure reliable, consistent, and confirmed results. The results demonstrate that, even with an extensive data set, including anthropometric, clinical, neuromuscular, genetic, and sophisticated 3-dimensional biomechanical measurements, ACL injury prediction was poor (mean AUC-ROC, 0.63 for the best method). Thus, while statistically significant predictive ability was discovered, it remained too low for use in clinical risk assessment. Importantly, our results indicate that the included variables, even those identified as risk factors in previous explanatory studies, are not able to predict ACL injuries in practice. Nevertheless, associations in this prospective data set may still be valuable for understanding injury causation, but further analysis on variables is outside the scope of this paper.

### Methodological Considerations

The wide range of AUC-ROC values across repetitions is notable (Table 1) and demonstrates that the use of a single random cross-validation split can lead to highly varying interpretations based on the same data and analyses, even with the current data set, which is by far the largest prospective cohort study for ACL injury in a team/ball sport. This variability was clearly visible in the results of Ruddy et al^44^ as well. As cross-validation is based on randomly splitting the data into k sets, each model is trained on a different, random subsample of data and results vary. Repeated analysis can be used to handle and investigate the variation in results and reach more robust and reliable estimates for the data. Utilizing a sufficient number of repetitions is essential for obtaining a reliable estimate (eg, average AUC-ROC) for the predictive performance. Additionally, noise in data introduces randomness in results as methods might capture the noise in prediction. Noise is inevitable in any real-world data,^15^ and assessing the significance of results is especially important with small data sets or with lower-performance results^8^ to make sure they reflect a truly present phenomenon. Our results were confirmed with permutation tests as suggested in Combrisson and Jerbi^8^ and Jauhiainen et al,^19^ and despite relatively low predictive performance, there was predictive ability since the results were significantly above chance level. This confirms the presence of true phenomena, and since these relationships were captured by all models, we can be relatively confident in these results.

Importantly, studies should also report predictive performance estimates for test and/or validation data to make reliable interpretations and rule out chance results. In our study, for 3 of the methods—namely, random forest and SVMs—the training AUC-ROC was noticeably higher than the test AUC-ROC. In general, random forests should be resilient to overfitting, as the combination of multiple decision trees reduces the variance of individual trees.^4^ With a hundred trained trees and the minimum leaf size of 50, this training AUC-ROC was surprisingly high, as more trees as well as larger minimum leaf size values should reduce overfitting.^4,14^ With the SVMs, the *box constraint* parameter can be used to control overfitting in MATLAB so that larger values lead to fewer support vectors. Looking at the parameter values chosen by optimization, the values seem relatively high (in the level of hundreds from 0.001 to 1000) for both SVMs, meaning the separation between classes remains simpler and smoother instead of overfitting. Thus, parameter selection for all methods seems appropriate despite high training AUC-ROCs. Previous studies show that high or near-perfect training AUC-ROC values do not cause a generalization problem with classifiers used in the current study, that is, random forest and SVM.^2,9^ Additionally, regularization seems to acceptably control overfitting of the logistic regression in our results, while the test AUC-ROC values are very similar compared with the other methods. This indicates that the predictive performance of our models was likely not largely affected by the high training AUC-ROC values.

The use of imbalance handling techniques before prediction did not improve the predictive performance. This could possibly be because of existing samples not being separable to begin with, in which case any resampling techniques would naturally not improve prediction. However, our AUC-ROC values were significantly higher than chance, indicating that some class separation is present in the data. In the studies by Ruddy et al^44^ and López-Valenciano et al,^25^ the use of SMOTE did not improve injury prediction, but random undersampling yielded slightly better results in the study by López-Valenciano et al. It seems that more studies are needed to assess the effect and necessity of imbalance handling in sport injury prediction.

### Using ML for Predicting Sport Injuries: Current Status

Recently, there have been a few examples of using ML approaches to predict sports injuries from data. Ruddy et al^44^ tested the predictive ability of previously recognized hamstring strain injury risk factors in 2 data sets with 186 and 176 elite Australian footballers and found them to have a failed predictive power (median AUC-ROCs, 0.58 and 0.52). Jauhiainen et al^19^ predicted knee and ankle injuries from a data set with 314 young basketball and floorball players and obtained an AUC-ROC value of 0.65. López-Valenciano et al^25^ used screening data with personal, psychological, and neuromuscular measures to predict muscle injuries in 122 male professional soccer and handball players and found AUC-ROC values up to 0.747. Their study, however, did not assess the stability of random k-fold division and only reported results from a singular repetition. Considering the randomness from cross-validation, class imbalance (23.7% injured), and extensive testing of different approaches, the possibility of chance findings would be important to consider in their results.^18^ Rommers et al^42^ achieved both precision (fraction of true injuries among those predicted as injuries) and recall (fraction of injuries that were correctly predicted) of 0.85 when predicting acute and overuse injuries in 734 elite youth soccer players with 20% holdout test data. This study was different from all previous studies in its age range (11.7 ± 1.7 years) as well as the fact that no class imbalance existed with 368 injured players (50.1% of players). They reported that the 5 most important variables that predict injury were anthropometric measures. The results indicate that injuries are possibly easier to predict accurately among teenagers during the growth spurt as well as if a more balanced data set can be collected. Taborri et al^51^ predicted “ACL injury risk” (Landing Error Scoring System [LESS] score, >5)^30^ with data from inertial sensors and optoelectronic bars and obtained an accuracy and F1 score of 0.96 and 95%, respectively. However, the LESS score has been shown to have no association with ACL injury with biomechanical data,^47^ and its validity with wearable data has not been investigated previously. In addition, their study had a small sample size (N = 39) and did not assess the stability of random k-fold division and the possibility of chance results.

### Using ML for Predicting Sports Injuries: Future Considerations and Conclusions

Considering the scale of different classification and preprocessing methods investigated in our analyses, it is possible that other tests or variables than the ones we have measured would be better for predicting ACL injuries. It has been suggested that the VDJ test is not a suitable screening test for ACL injury in female soccer and handball players.^21,32,38,49^ Additionally, training and match loads were not recorded in our data. It is also possible that 1 single screening test is not suitable for injury prediction, as baseline variables might change during follow-up.^28^ However, in the current data set it has previously been reported that changes in landing biomechanics were minor and that the consistency was high 2 years apart.^21,49^ It has been suggested that future studies exploit more continuous monitoring of athletes and consider short-term changes in physical variables and training loads.^19^ Recent studies indicate that wearable sensors and smartphone applications could be used to replace traditional laboratory motion data collection.^39^ Additionally, there are predictive studies showing potential in continuous monitoring and wearable sensors in injury prediction.^10,43^ Rossi et al^43^ predicted noncontact injuries in the next training session or game based on recent training load measured by wearable sensors in 26 professional male soccer players. They repeated the analysis 10,000 times to assess the stability with respect to fold divisions and achieved an AUC-ROC value of 0.78 ± 0.12. While their results are promising, the study is limited by a relatively small sample size and large class imbalance (in training data, 279 noninjury examples vs 7 injury examples). Dower et al^10^ predicted risk of soft tissue injuries in Australian rules football with GPS data. They achieved AUC-ROC values between 0.75 and 0.80 with repeated tests to ensure the stability of k-fold results.

## Conclusion

Despite analyzing a large prospective data set with extensive anthropometric, clinical, genetic, neuromuscular, and biomechanical measurements, using a variety of ML methods, the predictive ability was too low for ACL injury risk assessment in clinical practice. Therefore, further studies are needed to investigate what type of data and ML approaches should be used for more accurate injury prediction.

## Supplemental Material

sj-pdf-1-ajs-10.1177_03635465221112095 – Supplemental material for Predicting ACL Injury Using Machine Learning on Data From an Extensive Screening Test Battery of 880 Female Elite Athletes Click here for additional data file. Supplemental material, sj-pdf-1-ajs-10.1177_03635465221112095 for Predicting ACL Injury Using Machine Learning on Data From an Extensive Screening Test Battery of 880 Female Elite Athletes by Susanne Jauhiainen, Jukka-Pekka Kauppi, Tron Krosshaug, Roald Bahr, Julia Bartsch and Sami Äyrämö in The American Journal of Sports Medicine
