# Supplementary material for: Predicting ACL Injury Using Machine Learning on Data From an Extensive Screening Test Battery of 880 Female Elite Athletes
Source: Am J Sports Med. 2022 Aug 19;50(11):2917–24. doi: 10.1177/03635465221112095 (PMC9442771; doi:10.1177/03635465221112095)
Supplement: sj-pdf-1-ajs-10.1177_03635465221112095 – Supplemental material for Predicting ACL Injury Using Machine Learning on Data From an Extensive Screening Test Battery of 880 Female Elite Athletes [file sj-pdf-1-ajs-10.1177_03635465221112095.pdf]

Table A1: Variables included in predictive analysis. Those with grey background were measured for both left and right side (and transformed to dominant/non-dominant for analyses). For more detailed description, see [1] *and supplementary material therein. VDJ and CUT stand for the 3D motion analysis for vertical drop jump and cutting tasks, respectively.*

**Variable name**	**Test**	**Variable description**
AGE TESTED	Questionnaire	Age when tested (yr)
BODYMASS	Anthropometrics	Bodymass (kg)
HEIGHT	Anthropometrics	Height (cm)
AGE STARTED ELITE PLAY	Questionnaire	Age when started to play in elite level (yr)
NUMBER OF SEASONS ELITE PLAY	Questionnaire	How many seasons have played at the elite level
MATCH HOURS AVG WEEK	Questionnaire	Average match hours per week (n)
PREVIOUS ACL	Questionnaire	Do you have a previous ACL injury (y/n)
FAMILY ACL HISTORY	Questionnaire	Have your siblings or parents had and ACL injury (y/n)
CURRENT ACL PREVENTION	Questionnaire	Current participation in an ACL prevention program (y/n)
LEGPRESS MAX	Strength	Leg press one repetition maximum (kg)
KT1000 PREDEF PULL MM	Joint laxity	KT 1000 with predefined pull (mm)
KT1000 MANUAL PULL MM	Joint laxity	KT 1000 manual pull until natural stop (mm)
HAM MOBILITY DEGREES	Joint laxity	Hamstring mobility (deg)
GENU RECURVATUM DEGREES	Joint anatomy	Left genu recurvatum (deg)
HIP ANTEVERSION DEGREES	Joint anatomy	Left hip anteversion (deg)
KNEE VALGUS STATIC	Static calibration recording	Static valgus right knee
PELVIS FORWARD TILT STATIC	Static calibration recording	Anterior pelvic tilt, relative to the floor (deg)
PELVIS L TILT STATIC	Static calibration recording	Sideways tilt (to the left), relative to the floor (deg)
PELVIS L ROT STATIC	Static calibration recording	Sideways rotation (to the left), relative to the anterior global axis (deg)
PELVIS WIDTH	Static calibration recording	Distance left-right ASIS (m)
FEMUR LENGTH	Static calibration recording	Femoral length, distance between the hip and knee joint centers (m)
TIBIA LENGTH	Static calibration recording	Tibial length, distance between the knee and ankle joint centers (m)
FEMUR CONDYLE WIDTH	Anthropometrics	Width of femur condyle (m)
TIBIA CONDYLE WIDTH	Anthropometrics	Width of tibia condyle (m)
LEG LENGTH	3D-analysis	Leg length, from the hip joint center to the floor (m)
QUAD BEST	Strength	Peak torque, isometric knee extension (Nm)
HAM BEST	Strength	Peak torque, isometric knee flexion (Nm)
H Q RELATION	Strength	H:Q peak torque ratio (%)
SINGLELEG SQUAT HIP	Subjective rating	Hip control single leg squat (poor, fair, good)
SINGLELEG SQUAT KNEE	Subjective rating	Knee control single leg squat (poor, fair, good)
SINGLELEG DROPJUMP HIP	Subjective rating	Hip control single leg drop jump (poor, fair, good)
SINGLELEG DROPJUMP KNEE	Subjective rating	Knee control single leg drop jump (poor, fair, good)

DROPJUMP BILATERAL	Subjective rating	Knee and hip control vertical drop jump (poor, fair, good)
ANTEROLATERAL CM	Balance	Anterolateral (cm); Star Reach. Average of three trials
MEDIOLATERAL CM	Balance	Mediolateral (cm); Star Reach. Average of three trials
POSTEROLATERAL CM	Balance	Posterolateral (cm); Star Reach. Average of three trials
HIP ABDUCTION KG	Strength	Hip abduction strength (kg); isometric
NAVICULARDROP	Anthropometrics	Navicular drop, average of two trials (m)
GL INDEX	Anthropometrics	Generalized joint laxity. Joint laxity measured at the trunk, the fifth fingers, thumbs, elbows, and knees. The score of four points or more on a scale of 0–9 indicates generalized joint laxity.
JUMP HIP FLEX IC	VDJ	Hip flexion at initial contact (deg)
JUMP HIP FLEX MAX	VDJ	Maximal hip flexion during contact (deg)
JUMP HIP ABD IC	VDJ	Hip abduction at initial contact (deg)
JUMP HIP ABD MAX	VDJ	Maximal hip abduction during contact (deg)
JUMP HIP ROT IC	VDJ	Hip int. rotation at initial contact (deg)
JUMP KNEE FLEX IC	VDJ	Knee flexion at initial contact (deg)
JUMP KNEE FLEX MAX	VDJ	Maximal knee flexion during contact (deg)
JUMP KNEE VALGUS IC	VDJ	Knee valgus at initial contact (deg)
JUMP KNEE VALGUS MAX	VDJ	Maximal knee valgus during contact (deg)
JUMP KNEE ROT IC	VDJ	Knee int. rotation at initial contact (deg)
JUMP ANKLE PLFLEX IC	VDJ	Ankle plantar flexion at initial contact (deg)
JUMP ANKLE PLFLEX MAX	VDJ	Maximal ankle plantar flexion during contact (deg)
JUMP ANKLE INV IC	VDJ	Ankle inversion at initial contact (deg)
JUMP ANKLE INV MAX	VDJ	Maximal ankle inversion during contact (deg)
JUMP ANKLE ROT IC	VDJ	Ankle int. rotation at initial contact (deg)
JUMP HIP MOM FLEX MAX	VDJ	Maximal hip flexion moment during contact (deg)
JUMP HIP MOM FLEX MAX100	VDJ	Maximal hip flexion moment during first 100 ms of contact (deg)
JUMP HIP MOM ABD MAX	VDJ	Maximal hip abduction moment during contact (deg)
JUMP HIP MOM ABD MAX100	VDJ	Maximal hip abduction moment during first 100 ms of contact (deg)
JUMP KNEE MOM FLEX MAX	VDJ	Maximal knee flexion moment during contact (deg)
JUMP KNEE MOM FLEX MAX100	VDJ	Maximal knee flexion moment during first 100 ms of contact (deg)
JUMP KNEE MOM ABD MAX	VDJ	Maximal knee abduction moment during contact (deg)
JUMP KNEE MOM ABD MAX100	VDJ	Maximal knee abduction moment during first 100 ms of contact (deg)
JUMP ANKLE MOM DFLEX MAX	VDJ	Maximal ankle dorsiflexion moment during contact (deg)
JUMP ANKLE MOM DFLEX MAX100	VDJ	Maximal ankle dorsiflexion moment during first 100 ms of contact (deg)
JUMP ANKLE MOM INV MAX	VDJ	Maximal ankle inversion moment during contact (deg)
JUMP ANKLE MOM INV MAX100	VDJ	Maximal ankle inversion moment during first 100 ms of contact (deg)
JUMP GRF VERT MAX	VDJ	Maximal vertical ground reaction force (deg)
JUMP GRF VERT TIME MAX	VDJ	Time for maximal vertical ground reaction force (ms)

JUMP GRF MED MAX	VDJ	Maximal medial ground reaction force (N)
JUMP GRF MED TIME MAX	VDJ	Time for maximal medial ground reaction force (N)
JUMP GRF POST MAX	VDJ	Maximal posterior ground reaction force (N)
JUMP GRF POST TIME MAX	VDJ	Time for maximal posterior ground reaction force (ms)
JUMP HIP FLEX TIME MAX	VDJ	Time for maximal hip flexion (ms)
JUMP KNEE FLEX TIME MAX	VDJ	Time for maximal knee flexion (ms)
JUMP KNEE VALGUS TIME MAX	VDJ	Time for maximal knee valgus (ms)
JUMP ANKLE PLFLEX TIME MAX	VDJ	Time for maximal ankle plantar flexion (ms)
JUMP HIP MOM FLEX TIME MAX	VDJ	Time for maximal hip flexion moment (ms)
JUMP HIP MOM ABD TIME MAX	VDJ	Time for maximal hip abduction moment (ms)
JUMP KNEE MOM FLEX TIME MAX	VDJ	Time for maximal knee flexion moment (ms)
JUMP KNEE MOM ABD TIME MAX	VDJ	Time for maximal knee abduction moment (ms)
JUMP IMPULSE	VDJ	Total impulse from the ground (Ns)
JUMP FPPA IC	VDJ	Frontal plane projection angle at initial contact (deg)
JUMP FPPA MAX	VDJ	Maximal frontal plane projection angle during contact (deg)
JUMP COM HEIGHT IC	VDJ	Center of mass height at initial contact (m)
JUMP COM HEIGHT MIN	VDJ	Center of mass minimum height during the stance phase (m)
JUMP COM HEIGHT TIME MIN	VDJ	Time after initial contact for the center of mass minimum height during the stance phase (ms)
JUMP COM VERTICAL SPEED IC	VDJ	Center of mass vertical velocity at initial contact (m/s)
JUMP TORSO FLEX IC	VDJ	Torso flexion relative to the pelvis, at initial contact (deg)
JUMP HEIGHT	VDJ	Center of mass elevation, relative to its vertical position while standing (m)
CUT HIP FLEX IC	CUT	Hip flexion at initial contact (deg)
CUT HIP FLEX MAX	CUT	Maximal hip flexion during contact (deg)
CUT HIP ABD IC	CUT	Hip abduction at initial contact (deg)
CUT HIP ABD MAX	CUT	Maximal hip abduction during contact (deg)
CUT HIP ROT IC	CUT	Hip int. rotation at initial contact (deg)
CUT KNEE FLEX IC	CUT	Knee flexion at initial contact (deg)
CUT KNEE FLEX MAX	CUT	Maximal knee flexion during contact (deg)
CUT KNEE VALGUS IC	CUT	Knee valgus at initial contact (deg)
CUT KNEE VALGUS MAX	CUT	Maximal knee valgus during contact (deg)
CUT KNEE ROT IC	CUT	Knee int. rotation at initial contact (deg)
CUT ANKLE PLFLEX IC	CUT	Ankle plantar flexion at initial contact (deg)
CUT ANKLE PLFLEX MAX	CUT	Maximal ankle plantar flexion during contact (deg)
CUT ANKLE INV IC	CUT	Ankle inversion at initial contact (deg)
CUT ANKLE INV MAX	CUT	Maximal ankle inversion during contact (deg)
CUT ANKLE ROT IC	CUT	Ankle int. rotation at initial contact (deg)
CUT HIP MOM FLEX MAX	CUT	Maximal hip flexion moment during contact (deg)

CUT HIP MOM FLEX MAX100	CUT	Maximal hip flexion moment during first 100 ms of contact (deg)
CUT HIP MOM ABD MAX	CUT	Maximal hip abduction moment during contact (deg)
CUT HIP MOM ABD MAX100	CUT	Maximal hip abduction moment during first 100 ms of contact (deg)
CUT KNEE MOM FLEX MAX	CUT	Maximal knee flexion moment during contact (deg)
CUT KNEE MOM FLEX MAX100	CUT	Maximal knee flexion moment during first 100 ms of contact (deg)
CUT KNEE MOM ABD MAX	CUT	Maximal knee abduction moment during contact (deg)
CUT KNEE MOM ABD MAX100	CUT	Maximal knee abduction moment during first 100 ms of contact (deg)
CUT ANKLE MOM DFLEX MAX	CUT	Maximal ankle dorsiflexion moment during contact (deg)
CUT ANKLE MOM DFLEX MAX100	CUT	Maximal ankle dorsiflexion moment during first 100 ms of contact (deg)
CUT ANKLE MOM INV MAX	CUT	Maximal ankle inversion moment during contact (deg)
CUT ANKLE MOM INV MAX100	CUT	Maximal ankle inversion moment during first 100 ms of contact (deg)
CUT GRF VERT MAX	CUT	Maximal vertical ground reaction force (N)
CUT GRF VERT TIME MAX	CUT	Time for maximal vertical ground reaction force (ms)
CUT GRF MED MAX	CUT	Maximal medial ground reaction force (N)
CUT GRF MED TIME MAX	CUT	Time for maximal medial ground reaction force (N)
CUT GRF POST MAX	CUT	Maximal posterior ground reaction force (N)
CUT GRF POST TIME MAX	CUT	Time for maximal posterior ground reaction force (ms)
CUT HIP FLEX TIME MAX	CUT	Time for maximal hip flexion (ms)
CUT KNEE FLEX TIME MAX	CUT	Time for maximal knee flexion (ms)
CUT KNEE VALGUS TIME MAX	CUT	Time for maximal knee valgus (ms)
CUT ANKLE PLFLEX TIME MAX	CUT	Time for maximal ankle plantar flexion (ms)
CUT HIP MOM FLEX TIME MAX	CUT	Time for maximal hip flexion moment (ms)
CUT HIP MOM ABD TIME MAX	CUT	Time for maximal hip abduction moment (ms)
CUT KNEE MOM FLEX TIME MAX	CUT	Time for maximal knee flexion moment (ms)
CUT KNEE MOM ABD TIME MAX	CUT	Time for maximal knee abduction moment (ms)
CUT IMPULSE	CUT	Impulse from the ground (Ns)
CUT TORSO PELVIS FLEX IC	CUT	Torso flexion relative to the pelvis at initial contact (deg)
CUT TORSO PELVIS LATFLEX IC	CUT	Torso lateral flexion relative to the pelvis at initial contact (deg)
CUT TORSO PELVIS LROT IC	CUT	Torso left rotation relative to the pelvis at initial contact (deg)
CUT TORSO GROUND FLEX IC	CUT	Torso flexion relative to the ground and speed direction at initial contact (deg)
CUT TORSO GROUND LATFLEX IC	CUT	Torso lateral flexion relative to the ground and speed direction at initial contact (deg)
CUT TORSO GROUND LROT IC	CUT	Torso left rotation relative to the ground and speed direction at initial contact (deg)
CUT TORSO LROT SPEED IC	CUT	Torso left rotational velocity relative to the ground and speed direction at initial contact (deg/s)
CUT STANCE TIME	CUT	Contact time (ms)
CUT CUTTING ANGLE	CUT	Cuting angle (deg)
CUT APPROACH SPEED IC	CUT	Approach speed (m/s)
CUT FOOT RROT IC	CUT	Foot progression angle, relative to the center of mass speed direction, at initial contact (deg)

CUT TOE LANDING IC	CUT	Foot sagittal plane inclination relative to the ground (deg)
CUT CUT WIDTH COM IC	CUT	Angle between the line from center of mass to its projection on the ground and the line from the center of mass to the foot center of pressure. Projected on the (frontal) plane perpendicular to the approach speed vector (deg)
CUT CUT DEPTH COM IC	CUT	Angle between the line from the center of mass to its projection on the ground and the line from the center of mass to the foot center of pressure. Projected on the (sagittal) plane containing the approach speed vector (deg)
CUT CUT WIDTH PELVIS IC	CUT	Angle between the line from the center of mass to its projection on the ground and the line from the center of mass to the foot center of pressure. Projected on the frontal plane of the pelvis (deg)
CUT CUT DEPTH PELVIS IC	CUT	Angle between the line from the center of mass to its projection on the ground and the line from the center of mass to the foot center of pressure. Projected on the sagittal plane of the pelvis (deg)
CUT MOMENTARM SAGITTAL 40	CUT	Moment arm of the ground reaction force vector to the knee, projected into the local sagittal plane, 40ms after initial contact (m)
CUT MOMENTARM FRONTAL 40	CUT	Moment arm of the ground reaction force vector to the knee, projected into the local frontal plane, 40ms after initial contact (m)
CUT SIMPLEMOM FRONTAL MAXMOM	CUT	Peak simple knee abduction moment, calculated as the product of the ground reaction force magnitude and moment arm in the frontal plane (Nm)
CUT MOMENTARM FRONTAL MAXMOM	CUT	Moment arm of the ground reaction force vector to the knee, projected into the local frontal plane, at peak simple frontal moment (Nm)
CUT GRF FILT MAXMOM	CUT	Filtered ground reaction force, at peak simple frontal moment (N)

1. Pasanen K, Rossi MT, Parkkari J, et al. Predictors of lower extremity injuries in team sports (PROFITS-study): a study protocol. *BMJ open Sport Exerc Med*. 2015;1:e000076.
